# Recent developments in near-infrared spectroscopy (NIRS) for the assessment of local skeletal muscle microvascular function and capacity to utilise oxygen

**DOI:** 10.1016/j.artres.2016.09.001

**Published:** 2016-12

**Authors:** Siana Jones, Scott T. Chiesa, Nishi Chaturvedi, Alun D. Hughes

**Affiliations:** Institute of Cardiovascular Science, University College London, WC1E 6BT, UK

**Keywords:** Near-infrared spectroscopy, Skeletal muscle, Exercise

## Abstract

**Purpose of review:**

Continuous wave near infrared spectroscopy (CW NIRS) provides non-invasive technology to measure relative changes in oxy- and deoxy-haemoglobin in a dynamic environment. This allows determination of local skeletal muscle O_2_ saturation, muscle oxygen consumption ($\dot{V}{\text{O}}_{2}$) and blood flow. This article provides a brief overview of the use of CW NIRS to measure exercise-limiting factors in skeletal muscle.

**Recent findings:**

NIRS parameters that measure O_2_ delivery and capacity to utilise O_2_ in the muscle have been developed based on response to physiological interventions and exercise. NIRS has good reproducibility and agreement with gold standard techniques and can be used in clinical populations where muscle oxidative capacity or oxygen delivery (or both) are impaired. CW NIRS has limitations including: the unknown contribution of myoglobin to the overall signals, the impact of adipose tissue thickness, skin perfusion during exercise, and variations in skin pigmentation. These, in the main, can be circumvented through appropriate study design or measurement of absolute tissue saturation.

**Summary:**

CW NIRS can assess skeletal muscle O_2_ delivery and utilisation without the use of expensive or invasive procedures and is useable in large population-based samples, including older adults.

## Highlights

• An overview of CW NIRS to measure O_2_ utilisation and delivery is presented.
• CW NIRS is cheap, non-invasive, portable and useable in population-based samples.
• It is useful for understanding underlying mechanisms of deterioration in capacity.

## Introduction

Near Infrared Spectroscopy (NIRS) has been used to assess tissue oxygenation (saturation/perfusion), local O_2_ consumption ($\dot{V}{\text{O}}_{2}$), a measure of oxidative metabolism, and blood flow in various human tissues including the brain and skeletal muscle.^1^, ^2^ Combining NIRS with simple physiological interventions, such as venous or arterial occlusions, allows quantitative measurements to be made from skeletal muscle. This provides a tool for assessing two major determinants of the capacity of muscles to exercise: O_2_ delivery and O_2_ utilisation. The non-invasive nature of NIRS makes it an appealing technique for use in a dynamic environment and for activities of daily living.

Previous review articles have presented a historical overview of NIRS development.^1^, ^2^ Between 2007 and 2011 three reviews focused on oxidative metabolism and mitochondrial function in skeletal muscle.^3^, ^4^, ^5^ A recent article addressed NIRS instrumentation including its progression to allow near-infrared cerebral imaging (NIRI) in 2D (topography) and 3D (tomography).^6^ The extent to which the field has been reviewed highlights the rapid development of instrumentation and methodology and also the importance of continuous re-evaluation of NIRS specific to its application.

The purpose of this article is to provide a brief synopsis of recent development in continuous wave (CW) NIRS measurements from skeletal muscle. Discussion centres on its applicability for use in a clinical environment in the context of exercise testing as a means to determine the underlying mechanisms relating to reduced exercise capacity in unhealthy or older individuals. The major limitations of NIRS, suggested methods of circumventing them and assumptions regarding signal components are reviewed. An in depth description of NIRS instrumentation and manufacturer specific variation is not covered as this would be beyond the scope of a short review and can be found elsewhere.^1^, ^6^

### NIRS technology

Near-infrared light can penetrate biological tissues with less scattering and absorption than visible light and consequently offers advantages for imaging and quantitative measurements.^7^ In its simplest form, a NIRS device consists of a light-source emitting 2 or more wavelengths of light in the near-infrared range (650–1000 nm) into the tissue of interest and a detector placed at a known distance from the source(s). The chromophores haemoglobin (Hb) and myoglobin (Mb) are oxygen carriers in blood and skeletal myocytes respectively and their absorbance of near infra-red light differs depending on whether they are in an oxygenated or deoxygenated state. Unfortunately the spectral absorbance of the oxy- and deoxy-Hb and Mb is almost indistinguishable^8^ so attenuation by skeletal muscle is attributable to both chromophores. Estimates of relative contribution from Hb and Mb to the NIRS signal are conflicting: Hb has been proposed to contribute as much as ∼90%^9^, ^10^ or as little as ∼10–20%^11^, ^12^, ^13^ to the signal. A recent paper by Davis and Barstow ^14^ critically reviewed the topic and estimated the likely contributions of Hb and Mb to NIRS signals based on anatomical and experimental data. They suggested that Mb is likely to contribute ∼50–70% of the NIRS signals at rest in many, but not all, mammalian skeletal muscles. They also estimated that the relative contribution from Mb was likely to increase during exercise.^14^ Lai et al. used a mathematical model of O_2_ transport, metabolism and distribution of blood volume in muscle and suggested that the Mb contribution is dynamic and varies in relation to blood flow.^15^ On the basis of simulations of muscle response under hypoxic and normoxic conditions Spires et al.^16^ suggested Mb would be more affected by reductions in blood flow than Hb and proposed that the Mb contribution to NIRS signals may therefore differ in disease.^16^

Relative oxy- and deoxy-Hb/Mb concentrations can be estimated from NIRS signals using the modified Beer-Lamberts Law.^6^ Algorithms for determining concentrations are discussed in detail elsewhere.^6^ The modified Beer-Lamberts Law allows only relative, not absolute, concentrations to be derived. This is because biological tissue is not homogenous, therefore the tissue's optical properties (the scattering and absorption coefficients) and the absolute path-length which the light travels cannot be determined using CW NIRS. Time and frequency resolved NIRS techniques include a measurement of the absolute optical path-length and the intensity of light at the detector. This allows absolute concentration to be measured^1^; however scattering (and hence optical path-length) may change as a result of some typical interventions (e.g. exercise) in skeletal muscle complicating the interpretation of NIRS data.^5^, ^17^

#### Multi-distance algorithms

Multi-distance algorithms measure from two or more tissue depths and incorporate the diffusion equation to allow a % absolute tissue saturation index (TSI; also termed tissue oxygenation index) to be calculated. Spatially resolved spectroscopy (SRS) and the self-calibrating (SC) method are the 2 main multi-distance approaches. The slope of light attenuation versus source-detector separation distance is determined, from which the absorption coefficient can be calculated by applying diffusion theory. The tissue is assumed to be homogeneous in these methods and the scattering coefficient is modelled according to the light wave-length. Thus, these methods are thought to account for the influence of superficial tissue layers; hence TSI values can be compared directly between subjects. A correction factor for TSI can be calculated by placing a third light source directly between the first and second source; attenuation at the middle distance should be intermediate between the shortest optode-detector distance and the longest (Personal communication: Portamon, Artinis Medical Systems, The Netherlands).

#### Signal components

**Figure 1:**
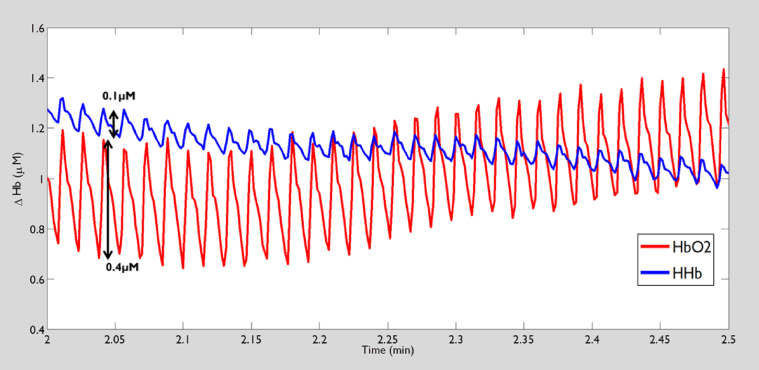
Example trace showing oxy (HbO_2_) and deoxy (HHb) haemoglobin signals measured in human lateral head of the gastrocnemius muscle under resting supine conditions. The cyclical oscillation of signal seen in the HbO_2_ and to a lesser extent in HHB signal corresponds with the cardiac cycle. Arrows indicate the magnitude of change in oxy (0.4 μM) and deoxy (0.1 μM) associated with an individual cardiac cycle.

Light passing into blood vessels >1 mm diameter should contribute little to NIRS signals as it will be almost completely absorbed. Oxy- and deoxy-Hb signals therefore represent Hb concentrations in blood vessels smaller than this (i.e. small arteries, arterioles, capillaries and venules).^18^, ^19^ It is generally assumed that the major part of the Hb-related NIRS signal arises from capillaries since these micro-vessels compose the largest portion of vascular volume in skeletal muscle. However, the synchronous cardiac pulsatile nature of the signal observed at rest in both oxy- and deoxy Hb signal from skeletal muscle (Fig. 1) suggests that at least some of signal arises from small arteries and larger arterioles where cardiac pulsatility has not dissipated.^20^ Since arteriolar and venular haemodynamics are not usually synchronous^20^, ^21^ the presence of simultaneous pulsatility in the oxy- and deoxy Hb signal in this example suggests some cross-talk between the signals, presumably due to the homogeneous medium assumption with the use of mean optical path length.^22^

Due to the stability of arterial O_2_ saturation, saturation changes that are unrelated to cardiac pulsatility or muscle contraction, are thought to predominantly represent changes in the venous compartment.^9^, ^18^ A hydrostatic change imposed by tilting from supine to upright results in venous filling and has been used to provide information about the compliance of the venous compartment.^23^, ^24^

## Limitations of NIRS in skeletal muscle

### Adipose tissue

Adipose tissue thickness at the site of measurement influences NIRS measurements through its effect on the scattering properties of the tissue.^25^ This has implications for the choice of measurement site. For example, thigh muscle (e.g. Vastus Lateralis) is a major working muscle for locomotion but the thickness of adipose tissue in the thigh may exceed 3 cm in people with diabetes.^26^ Although not directly related to locomotion, muscle in the forearm is often examined because adipose tissue is less thick here and occlusive cuffs can be easily applied above the elbow. For NIRS measurements in the lower limb of older individuals, the gastrocnemius may be a useful compromise since subcutaneous adipose thickness rarely exceeds 1 cm at this location.^27^

To allow comparison to be made between subjects with different adipose tissue thickness, a physiological calibration can be applied; this involves calibration of the relative concentration values to a normalized scale: a baseline is achieved by application of a total ischaemic occlusion until the oxy-Hb signal plateaus. The hyperaemic response, following cuff release, provides a functional maximum that can be used to scale responses.^28^ The hyperaemic response to 5 min occlusion of the brachial artery has recently been reported to show good intra-subject reproducibility.^29^ The technique may however be limited by poor subject acceptance of total occlusion, especially in older adults. Impairment of maximum hyperaemia due to vascular disease or incompressible calcified vessels may also limit this technique.

### Skin perfusion

During exercise skin perfusion may change significantly in response to the rise in body temperature^30^ and both oxy- and deoxy-Hb signals from skin may therefore confound the muscle signal. The contribution of skin to the NIRS signal has been considered minimal^9^; however, more recent studies indicate a more substantial contribution from skin blood flow.^31^ Use of spatially resolved techniques may reduce the impact of the cutaneous layer.^32^

### Melanin contribution

Melanin in the skin and Cytochrome also absorb light in the near infrared range. Wassenaar et al., 2005 described attenuation of light reflectance in direct relation to increased melanin in a small study.^33^ Technological developments addressing signal loss, such as increased signal intensity or improved detection, could be useful for reducing attenuation. Applying a physiological calibration (described above) to the signal allows inter-subject comparisons to be made with different levels of skin pigmentation.

### Heterogeneity of blood flow in the muscle

The heterogeneity of blood flow and O_2_ utilisation within the muscle can only be examined if multiple source-detector pairs are used.^34^, ^35^ There has been a recent increase in the number of skeletal muscle studies exploiting multi-channel instruments.^36^, ^37^, ^38^ Studies assessing the perfusion/utilisation relationship (matching of O_2_ delivery to requirement) provide a comprehensive assessment of skeletal muscle function but as the number of source-detector pairs increases data collection becomes more complex, and less portable. With development of improved analysis techniques and more portable complex devices, parameters for assessing ‘matching’ of O_2_ delivery and utilisation can be studied more readily. These studies also highlight the importance of consistent probe position within a study where inter-subject comparisons are made using a simple device with fewer source-detector pairs.

Despite these technical limitations, the non-invasive and cost effectiveness of CW NIRS makes it attractive. The technology has been developed in small, wireless instruments showing good intra- and inter-subject reproducibility^39^, ^40^ and recently, the TSI signal was found to be stable despite waterproofing and water submersion when a portable CW NIRS device was used during swimming.^41^

## NIRS applications in the clinical setting

Microvascular dysfunction is associated with a range of cardiometabolic diseases such as diabetes, hypertension and obesity. The cause-effect relationship in these disease processes remains unclear.^7^ It is therefore of interest to develop novel ways to assess microvascular function and better understand its relationship with macro-vascular function and metabolic disturbances. Due to the assumption that NIRS is capable of detecting signal only from small blood vessels, it provides researchers and clinicians with the opportunity to assess microvascular function^42^ in addition to a macro-vascular functional assessment obtained via more established methods.

NIRS has been used in various clinical populations where O_2_ delivery and/or utilisation of O_2_ are implicated in the disease process. Examples include muscle myopathies,^43^ diseases causing muscle atrophy,^44^ heart failure^45^, ^46^ and peripheral arterial (or vascular) disease^42^ (PAD).

## Assessment of skeletal muscle $\dot{V}{\text{O}}_{2}$

Diseases symptomatically characterised by breathlessness on exertion and muscle fatigue are severely debilitating for patients. An exercise test incorporating a measure of total body oxygen consumption ($\dot{V}{\text{O}}_{2\max}$), measured through breath-by-breath gas analysis, is commonly used to assess total body cardio-respiratory capacity; however this test does not necessarily identify the mechanism underlying functional limitations. Local assessments of O_2_ saturation and muscle oxidative capacity provide further mechanistic insights.

Oxidative capacity within skeletal muscle has previously been measured using techniques involving muscle biopsies or magnetic resonance spectroscopy (MRS).^47^ However, repeated biopsies are uncomfortable for participants and MRS is an expensive tool.

### Resting $\dot{V}{\text{O}}_{2}$

In the absence of changes in blood volume, NIRS signals represent the balance between O_2_ delivery and consumption. Applying an arterial or venous occlusion above the site of measurement is commonly used to provide a measure of local muscle $\dot{V}{\text{O}}_{2}$.^48^

**Figure 2:**
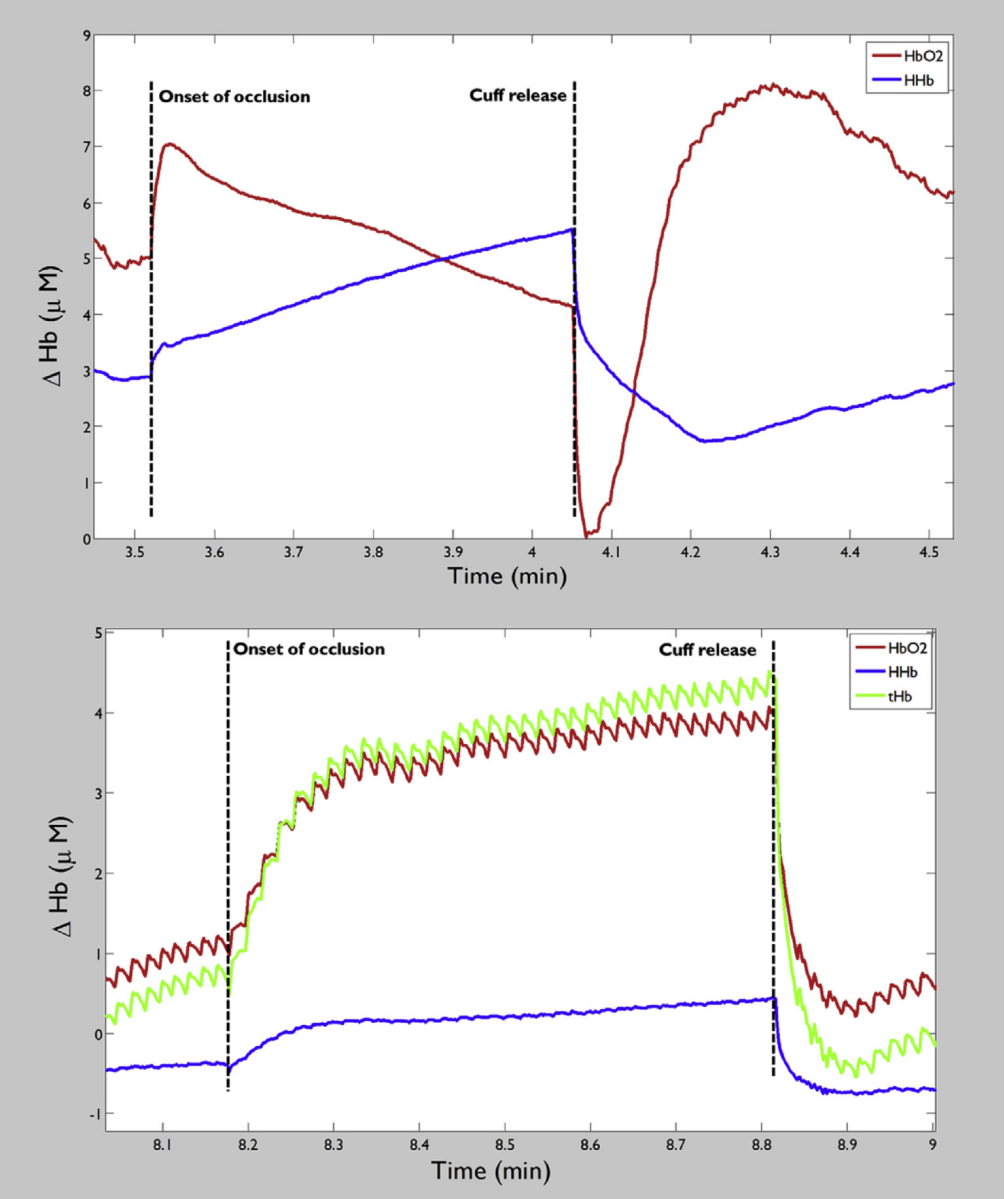
Example of an arterial (top panel) and a venous (bottom panel) occlusion for ∼30 s, vertical dashed lines show the onset of occlusion and release of cuff.

An arterial occlusion creates a closed circuit system, no blood flow in or out, so, in the absence of volume change between vascular compartments within the occluded tissue, the rate of decrease in oxy-Hb (or deoxy-Hb increase) represents the muscle $\dot{V}{\text{O}}_{2}$_,_ in μM/second (Fig. 2, top panel). During venous occlusion, arterial blood flow is maintained but venous outflow is obstructed until venous pressure exceeds the pressure in the occluding cuff (Fig. 2, bottom panel). In the early quasi-linear phase following inflation of the venous occlusion cuff the rate of increase in deoxy-Hb represents $\dot{V}{\text{O}}_{2}$^49^ and rate of increase in total Hb signal provides a measurement of resting blood flow.^50^

Studies comparing $\dot{V}{\text{O}}_{2}$ measured by venous and arterial occlusion have reported moderate correlations (Pearson's r = 0.647^48^ and Spearman's rho = 0.41^49^) but in both studies venous occlusion yielded estimates of resting $\dot{V}{\text{O}}_{2}$ that were ∼14–25% higher than values calculated following arterial occlusion. Venous occlusion derived $\dot{V}{\text{O}}_{2}$ values show higher variability^49^ and poorer reproducibility.^50^ The lower values measured by arterial occlusion may be explained by continued inflow of arterial blood during venous occlusion. Although arterial occlusions provide more accurate values the higher pressures necessary to completely restrict blood flow are less comfortable for the subject and in some subjects with incompressible arteries the assumption of complete occlusion may not obtain. Also, the assumption of no shifts in blood volume between vascular compartments during arterial occlusion may not hold and a method to correct for such volume shifts has been described recently.^51^

### $\dot{V}{\text{O}}_{2}$ during exercise

**Figure 3:**
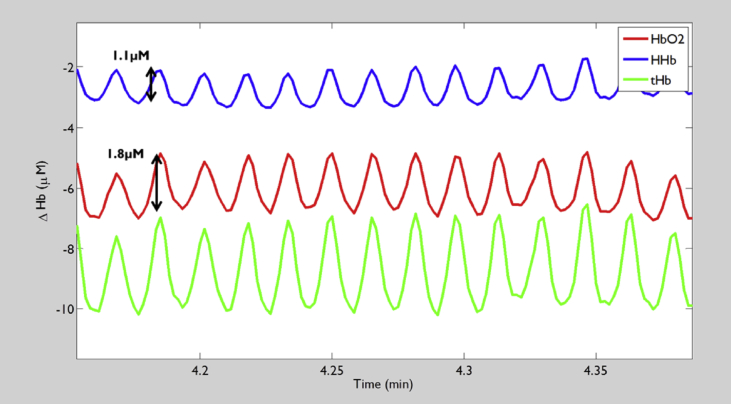
Example of cyclic changes in oxy haemoglobin (HbO_2_, red), deoxy haemoglobin (HHb, blue) and total haemoglobin (tHb, green) signals seen during rhythmic exercise (cross-training). (For interpretation of the references to colour in this figure legend, the reader is referred to the web version of this article.)

Exercise induces a large increase in muscle blood flow.^52^ During rhythmic exercise the muscle pump action acts on blood vessels to elicit volumetric shifts,^53^ these can be seen as cyclic changes in the oxy- and deoxy Hb signals, corresponding to stepping action (Fig. 3). It is not feasible to perform repeated arterial occlusions to estimate $\dot{V}{\text{O}}_{2}$ throughout exercise, so a simple alternative is to surmise that immediately post-exercise the $\dot{V}{\text{O}}_{2}$ determined via arterial occlusion is equivalent to the $\dot{V}{\text{O}}_{2}$ during the final stages of the exercising protocol.^54^

Recently, a model has been proposed that utilises the signal changes generated by the muscle pump during rhythmic cycling to generate values for instantaneous tissue saturation^55^ and instantaneous O_2_ utilisation.^56^ Oxygenation within the vessels affected by muscle pump action is lower during the contraction phase than during the relaxation phase. By determining ‘cyclic O_2_ saturation’^55^ and a net total Hb concentration change across an averaged pedal cycle (30 s sample), the number of O_2_ molecules within that net flow, can be determined. This instantaneous O_2_ consumption measurement is also appealing because muscle contraction should have minimal impact on blood flow within adipose tissue and the myoglobin contribution to the signal can be assumed constant.^56^ This approach has been used to demonstrate a significantly lower cyclic muscle O_2_ consumption measured during cycling at a low (40%) versus a high (110%) percentage peak aerobic power in athletic subjects.^56^ The instantaneous O_2_ consumption measurement assesses only the tissue interrogated by NIRS and subject to muscle pump action; it could have limitations in a scenario of more complex measurements using a greater number of source-detector pairs aiming to assess the saturation and consumption across the muscle. The technique also lacks rigorous testing in non-athletic subjects.

### Recovery of $\dot{V}{\text{O}}_{2}$ after exercise

A process of transient arterial occlusions in the immediate post-exercise period, originally described by Motobe et al in 2004,^57^ can be used to generate a time constant for recovery of muscle $\dot{V}{\text{O}}_{2}$ – a marker of skeletal muscle oxidative capacity. In healthy subjects the technique has shown good reproducibility that is uninfluenced by the type of exercise.^54^, ^58^ Good agreement has been demonstrated between NIRS derived recovery time constants and phosphocreatine (PCr) recovery time constants found with magnetic resonance spectroscopy (MRS)^59^ as well as good correlation with in vitro assessed oxidative capacity via muscle biopsy analysis.^60^

### The effect of training and disease on recovery of $\dot{V}{\text{O}}_{2}$ after exercise

Sensitivity of muscle $\dot{V}{\text{O}}_{2}$ recovery kinetics to athletic capacity has been demonstrated by several groups.^61^, ^62^ Brizendine et al. reported muscle $\dot{V}{\text{O}}_{2}$ recovery time constants of the Vastus Lateralis were nearly twice as fast in endurance athletes as non-athletic, age-matched controls.^61^ The effects of training and de-training on the recovery time constant of muscle $\dot{V}{\text{O}}_{2}$ measured by NIRS have also been shown in wrist flexor muscles.^63^ Re-oxygenation rate following exercise is faster in trained individuals than in non-trained controls^28^ and re-oxygenation rate can be improved by undertaking a training intervention.^64^ Re-oxygenation is slower in patients with diseases affecting O_2_ delivery, such as heart failure^65^ but recently, exercise training has been shown to improve this reduction.^45^

The studies discussed above support positive adaptions related to physical activity. The benefits gained from exercise-training can be attributed to improved O_2_ delivery (neovascularization) and/or increased mitochondrial capacity. However, benefits gained from exercise may differ depending on age or degree of disease and may not be comparable to the mechanisms of improved fitness in healthy individuals. It is therefore unclear if it is reasonable to compare studies including young athletic subjects with studies conducted in older adults.

## Post-occlusive reactive hyperaemia (PORH)

NIRS measurements during PORH can distinguish the muscle microvascular response of patients with PAD from healthy volunteers. PORH in PAD is characterized by smaller maximum hyperaemic responses, slower rates of reperfusion and recovery and consequently longer times to recovery and peak PORH.^66^ Reproducibility of NIRS measured PORH in the forearm, in healthy subjects, was recently shown to be good for all parameters measured.^29^

Bopp et al., 2014 found good correlation between micro and macro vascular PORH measured simultaneously by NIRS and brachial artery blood flow velocity by pulsed Doppler ultrasound (r = 0.91, p < 0.0001). They suggest using NIRS to quantify microvascular reactivity in the muscle is more suitable than comparisons made using previous methods, examining cutaneous vascular beds, which may be under different mechanisms of control.^67^ Gayda et al., 2014 reported that the microvascular assessment of PORH was more closely related to cardiovascular risk factors than the macro-vascular assessment using flow mediated dilatation (FMD) in a group comprised of healthy controls and patients with metabolic syndrome or coronary heart disease (CHD).^52^

## Exercise-induced blood volume changes in PAD

NIRS has been utilised during exercise in patients with PAD to examine tissue deoxygenation; the authors describe parameters generated from the area under the curve (AUC) of the Hb signals during the early stages of an incremental walking test (1.7–3.0 km/h walking speed).^12^ The AUC generated from all Hb signals, except for total Hb, were able to detect accentuated deoxygenation in PAD compared to healthy individuals. The same parameters were also used to investigate deoxygenation in PAD with and without diabetes in relation to delated reporting of claudication in participants with diabetes.^14^

## Microvascular venous compliance

Binzoni et al., 2000 first described the use of NIRS to assess microvascular compliance by imposing a passive orthostatic pressure challenge to the lower body and measuring the changes in Hb signals from the calf. They describe a method for generating a time constant that represents compliance of the venous (venular) compartment during the head-up tilt manoeuvre.^23^

More recently, Truijen et al., 2012 employed NIRS in complement to strain-gauge plethysmography to address the time-course of orthostatic changes in micro-vascular volume as well as total change in volume.^24^ The authors described a biphasic exponential model for microvascular filling during head-up tilt providing insight into the mechanism of increasing total leg volume prolonged standing described using classic strain gauge plethysmography.

## Conclusions

CW NIRS is a non-invasive, portable and relatively cheap way to measure local skeletal muscle O_2_ utilisation and delivery, which are both important determinants of functional capacity in exercising muscle. This provides the means to investigate pathophysiological mechanisms that limit exercise capacity as well as mechanisms of benefit resulting from exercise intervention. This is of value in a multitude of disease states, but also in the context of age related decline in exercise capacity. Understanding underlying mechanisms of deterioration in capacity would help identify optimal targets for intervention tailored to specific populations.

Physiological interventions such as arterial and venous occlusions generate quantitative values and have demonstrated good reproducibility, sensitivity to capacity and agreement with gold standard techniques. Future studies employing NIRS to measure oxygenation and/or muscle $\dot{V}{\text{O}}_{2}$ should be designed in light of the advantages and limitations of the technique.

## Funding sources

Institute of Cardiovascular Science UCL PhD stipend, and British Hearth Foundation CS/13/1/30327.

## Conflict of interest

None declared.
